# Supplementary material for: Comparative multiomics analysis of cell physiological state after culture in a basket bioreactor
Source: Sci Rep. 2022 Nov 23;12:20161. doi: 10.1038/s41598-022-24687-4 (PMC9686226; doi:10.1038/s41598-022-24687-4)
Supplement: Supplementary file 1 — Supplementary Information 1. [file 41598_2022_24687_MOESM1_ESM.zip › raw data/Transcriptomic raw data/6.Enrichment/1.GO/TE1vsTC1/DOWN/TE1vsTC1.down_GObar_mf.pdf]

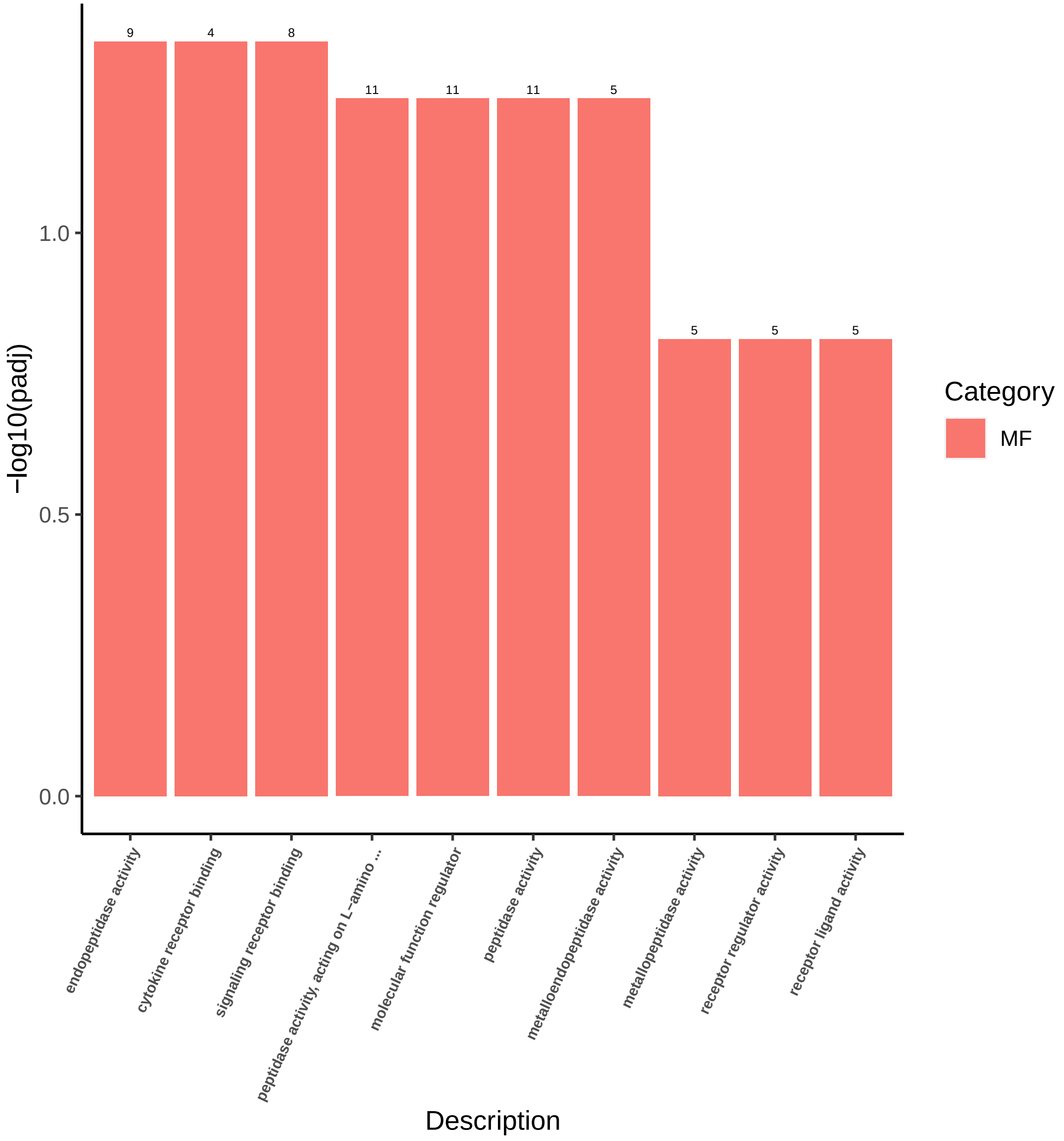

9
4
8
11
11
11
5
5
5
5
1.0
0.5
0.0
−log10(padj)
endopeptidase activity
cytokine receptor binding
signaling receptor binding
peptidase activity, acting on L−amino ...
molecular function regulator
peptidase activity
metalloendopeptidase activity
metallopeptidase activity
receptor regulator activity
receptor ligand activity
Description
Category
MF
